# The Effect of Blood Contamination on the Compressive Strength of Calcium-Enriched Mixture

**Published:** 2015-03

**Authors:** Alireza Adl, Nooshin Sadat Shojaee, Fereshte Sobhnamayan, Mohammadsaeed Hashemzade

**Affiliations:** a Dept. of Endodontics, School of Dentistry, Shiraz University of Medical Sciences, Shiraz, Iran.; b Student, Biomaterial Research Centre, School of Dentistry, Shiraz University of Medical Sciences, Shiraz, Iran.

**Keywords:** Blood contamination, Compressive strength, Calcium-enriched mixture, CEM

## Abstract

**Statement of the Problem:**

In clinical situations, Calcium-Enriched Mixture (CEM) comes into direct contact or even mixes with blood during or after placement.

**Purpose:**

The aim of this study was to evaluate the effect of blood contamination on the compressive strength of CEM.

**Materials and Method:**

Three experimental groups were included in this study. In the first group, CEM was mixed with distilled water and was exposed to normal saline (control group). In the second group, CEM cement was mixed with distilled water and then was exposed to blood. In the third group, CEM was mixed with and exposed to blood. Nine custom-made two-part split Plexiglas molds with five holes were used to form CEM samples for compressive strength testing (15 samples in each group). After 7 days of incubation, compressive bond strength testing was performed using a universal testing machine. Data were statistically analyzed using the Mann–Whitney U test with a significance level of *p*< 0.05.

**Results:**

Nine samples from group 3 were fractured during removal from the molds; the other six blocks had some cracks on their surfaces. Therefore, a compressive strength measurement was not obtainable for this group. No statistically significant difference was found between groups 1 and 2 (*p*> 0.05).

**Conclusion:**

It can be concluded that exposure to blood does not adversely affect the compressive strength of CEM, but incorporation of blood makes the cement very brittle.

## Introduction

Mineral trioxide aggregate (MTA) was introduced to endodontics in 1993 [1] and has been widely used in the repair of root perforations, [2-3] pulp capping, [4-5] and creating an apical barrier in teeth with open apices. [6] MTA has excellent sealing ability, [7-8] biocompatibility, [9] and the ability to stimulate osteoblasts; [10] in addition, it sets in wet environment. [11] However, its disadvantages include extended setting time, [11] poor handling, [12] and relatively high cost.

Calcium-Enriched Mixture (CEM) was introduced in 2006 as a root-end filling material. [13] This novel endodontic cement has similarities to MTA in terms of pH, increased flow, decreased working time and film thickness and is less costly. [14-15] CEM also has low cytotoxicity, excellent biocompatibility, and sealing ability. [13] It has been used in clinical situations such as pulp capping, pulpotomy, perforation repair, apical plug, root resorption, and periradicular surgery. [16-20]

Although in studies on its physical properties, CEM was not allowed to set in contact with blood;^13-14, 20-22^ in clinical situations, CEM comes into direct contact or even mixes with blood during or after placement. It has been shown that blood contamination can affect the physical properties of MTA.

An in vitro study showed that exposure to blood during setting has an adverse effect on marginal adaptation and the surface microstructure of MTA. [23]

Two separate studies evaluated the effect of blood contamination on the compressive strength and surface microstructure of MTA. It was concluded that blood incorporation into MTA structure reduced the compressive strength of the material. [24-25] Vanderweele *et al.* reported that contamination of perforation sites with blood before MTA application significantly reduced resistance to displacement. [26]

 Considering the similar applications of CEM and MTA, the question is whether contamination with blood influences the physical properties of CEM. Therefore, the present study was designed to evaluate the effect of blood contamination on the compressive strength of CEM. 

## Materials and Method

The material investigated was CEM (BioniqueDent; Tehran, Iran). Fresh human blood from a healthy volunteer in our research team was obtained by phlebotomy, performed by a trained individual in accordance with *Helsinki* ethical principles for medical research involving human subjects.

Nine custom-made two-part split Plexiglass molds were used in this experiment. Each mold had five holes with internal diameter of 4±0.1 mm and height of 6±0.1 mm.The molds were randomly allocated into three groups, prior to being filled with CEM.The groups (15 holes/sample in each) comprised:

Group 1: CEM mixed with distilled water and exposed to normal saline.
Group 2: CEM mixed with distilled water and exposed to blood.
Group 3: CEM mixed with blood and exposed to blood.

**Figure 1:**
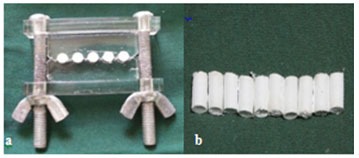
a: The experimental molds filled with the mixture of CEM and distilled water. b: The samples were removed from the molds.

CEM was prepared according to the manufacturer’s instructions and was then homogenized and positioned incrementally into the molds by amalgam carrier. After gentle packing and compacting with condensers, excess material was removed with wet cotton pellets (Figure 1).

In test groups 2 and 3, before placement of CEM cement, the molds were soaked in human blood and then removed to leave a small coating of blood on the internal surface of each hole in the molds. 

In group 3, CEM powder was prepared according to the manufacturer’s instructions on powder-to-liquid ratio, but blood was used instead of distilled water. After the placement of CEM, the molds were placed in Petri dishes containing appropriate medium in the lower part. The medium was normal saline in group 1 and heparinized blood in groups 2 and 3. Wet pieces of gauze were then placed above the molds but without coming into close contact with the CEM surface to produce fully saturated humidity. The plates were sealed and then placed in an incubator at 37°C.

After 7 days, the samples were removed from the incubator and the molds were split. The set CEM blocks were removed carefully by applying light force, taking care not to damage the CEM samples. After removal, the samples were evaluated for voids or cracks. 

**Figure 2:**
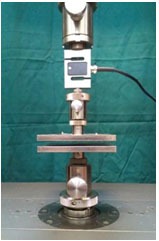
Universal testing machine for testing the compressive strength of the samples

To test the compressive strength, we placed the samples lengthwise between the platens of a universal testing machine (Zwick Roell Group; Germany) (Figure 2).

The samples were compressed at the speed of 1 mm/min, and the load at fracture was recorded in mega Pascals (MPa). The mean compressive strengths and standard deviation values were calculated for the groups and analyzed using the Mann–Whitney U test with a significance level of *p*< 0.05.

## Results

Nine samples from group 3 were fractured while being removed from the molds; the other six blocks had some cracks on their surfaces. Therefore, compressive strength measurement was not obtainable for this group. The means and standard deviations of the compressive strength of the groups are represented in Table 1.

**Table 1:** The means and standard deviations of compressive strengths (in MPa) of the experimental groups. Groups with the same letters were not significantly different.

Group	Mean	Standard deviation
Group 1	3.6000^a^	0.96749
Group 2	4.0220^a^	1.32033
Group 3	-	-

**Figure 3:**
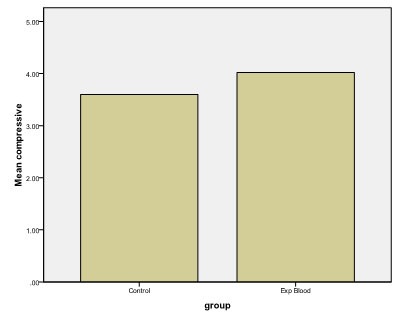
The compressive strengths of the experimental groups. (in MPa)

No statistically significant difference was found between groups 1 and 2 (*p*>0.05) (Figure 3).

## Discussion

In this study, the effect of blood contamination on the compressive strength of CEM was evaluated. Compressive strength is one of the indicators of the setting and strength of a material. [11, 27] Although mechanical tests are unable to reflect the clinical situation, they can show the effects of different mixing liquids and setting conditions on different types of cement.

According to ISO 9917-1 (2003) standards, for the compressive strength test, a split mold design (made of a material that will not be affected by the cement) has been advised. In studies on the compressive strength of MTA, one-piece plastic cylindrical molds, [28] one-piece polycarbonate cylindrical molds, [29] plastic split molds, [30] stainless steel split molds, [27] and one-piece borosilicate glass molds [24] have been used. In this study, two-part split Plexiglass molds were used to form CEM samples. A pilot study prior to this study showed that samples required a light force to allow removal. Although it has been reported that the setting time of CEM is shorter than that for MTA, [14] the aforementioned pilot study showed that the CEM samples were not solid before 7 days. Therefore, the compressive strength test was performed on day 7, while the studies on MTA evaluated compressive strength after 3 or 4 days. [24, 30-31]

CEM has been suggested for being used in pulp capping, pulpotomy, perforation repair, apical plug, root resorption, and periradicular surgery. [16-20] Therefore, in the majority of its clinical applications, CEM comes into contact and may mix with blood.

In this study, for simulation of the clinical situation whereby bleeding has been controlled, CEM was mixed with distilled water and exposed to fresh human blood. For simulation of situations with excessive bleeding in which blood may be incorporated into the material, CEM was first mixed with, and then exposed to blood. In the control group, CEM powder was mixed with distilled water and exposed to normal saline. It is noteworthy that mixing the endodontic cements with human blood as a model to replicate the clinical situation in which blood becomes incorporated into the cements has been previously reported. Nekoofar *et al.* mixed MTA with blood to investigate the effect of blood contamination on the compressive strength of two types of MTA. [24] Similarly in two separate studies, MTA was mixed with blood to evaluate its effect on the surface microhardness [32] and the microstructure of MTA. [33]

Despite using two-part split molds that required only light force for removal of the CEM samples, nine out of the 15 samples in group 3 were fractured during removal. Moreover, the other six samples had some cracks on their surfaces indicating that in the clinical situation, bleeding should be controlled before the placement of CEM to avoid unfavorable clinical outcomes.

In an *in vitro *study, Jasiczak and Zielinski demonstrated that mixing the powdered red blood cells with Portland cement reduced the compressive strength and increased the setting time of the cement. [34] Remadnia *et al.* also showed that hemoglobin or whole blood increased the porosity of Portland cement. [35] The findings of the present study, which demonstrated a decreased compressive strength of CEM mixed with blood, can be explained by the air entrapment effects of blood [34] and increased porosity of the cement. [35] Moreover, blood proteins may also affect the physical properties of CEM and interfere with hardening of the cement. Further studies should examine the effect of blood contamination on the structural porosity and formation of CEM crystals.

Although in the present study, exposure to blood did not reduce the compressive strength of CEM, Nekoofar *et al.* [24] reported that both exposure and mixing of MTA with blood could adversely affect its compressive strength. Since comparing the results of two separate studies with different methodologies is difficult, a new study is recommended for the comparison of the physical behaviors of MTA and CEM when contaminated with blood.

## Conclusion

Within the limitations of this *in vitro* study, it can be concluded that exposure to blood does not adversely affect the compressive strength of CEM; however, incorporation of blood makes the cement very brittle.
